# Assessment of communication technology and post-operative telephone surveillance during global urology mission

**DOI:** 10.1186/s13104-018-3256-2

**Published:** 2018-02-21

**Authors:** David E. Rapp, Andrew Colhoun, Jacqueline Morin, Timothy J. Bradford

**Affiliations:** 1 University of Virginia School of Medicine, Charlottesville, Virginia USA; 2 Virginia Commonwealth University School of Medicine, Richmond, Virginia USA; 3 Virginia Urology, Richmond, Virginia USA; 4 Global Surgical Expedition, 5829 Ascot Glen Drive, Glen Allen, VA 23059 USA

**Keywords:** Global health, Surgery, Follow-up

## Abstract

**Objective:**

Compliance with post-operative follow-up in the context of international surgical trips is often poor. The etiology of this problem is multifactorial and includes lack of local physician involvement, transportation costs, and work responsibilities. We aimed to better understand availability of communication technologies within Belize and use this information to improve follow-up after visiting surgical trips to a public hospital in Belize City. Accordingly, a 6-item questionnaire assessing access to communication technologies was completed by all patients undergoing evaluation by a visiting surgical team in 2014. Based on this data, a pilot program for patients undergoing surgery was instituted for subsequent missions (2015–2016) that included a 6-week post-operative telephone interview with a visiting physician located in the United States.

**Results:**

Fifty-four (n = 54) patients were assessed via survey with 89% responding that they had a mobile phone. Patients reported less access to home internet (59%), local internet (52%), and email (48%). Of 35 surgical patients undergoing surgery during 2 subsequent surgical trips, 18 (51%) were compliant with telephone interview at 6-week follow-up. Issues were identified in 3 (17%) patients that allowed for physician assistance. The cost per patient interview was $10 USD.

**Electronic supplementary material:**

The online version of this article (10.1186/s13104-018-3256-2) contains supplementary material, which is available to authorized users.

## Introduction

Surgical disease represents a significant problem in low- and middle-income countries (LMIC). Estimates suggest that 11% of disease worldwide is surgically treatable [1]. However, the poorest third of the global population receives only 3.5% of its surgical care [2]. International health initiatives have more recently been developed to address this need, including the World Health Organization (WHO) Global Initiative for Emergency and Essential Surgical Care [3].

Numerous international organizations have been created in an attempt to improve and deliver surgical care to the developing world and commonly deliver care through periodic surgical trips to countries in need. A notable challenge associated with this model is achieving longitudinal follow-up of patients after surgery. Following cleft lip and palate surgical trips, Foong et al. reported a 1-week follow-up rate of 60%, which decreased to 28% by 1-year follow-up [4]. Similar difficulties related to poor follow-up compliance after surgical trips are reported by other authors [5, 6].

Much of this difficulty centers around the nature of visiting surgical mission, whereby surgical teams perform a high volume of surgical cases over a short period of time. The problems associated with this fragmented model are well documented and include lack of involvement of local physicians and limited access to local health care practitioners with appropriate expertise, which often results in deficient long-term follow-up [7]. Given inadequate follow-up, visiting teams are prone to overestimate positive impact and underestimate adverse events associated with their surgeries [8].

Accordingly, research has focused on initiatives to improve follow-up using incentives as well as communication techniques. Travel subsidies, block appointments, and telephone reminders have all been shown to improve rates of follow-up after surgery [9–11]. More recently, mobile phone initiatives have been used to improve follow-up after surgery in developing countries [4].

Giving to Extremes Medical Missions (GTEMM) is a non-profit, tax-exempt charitable organization that sends surgical teams to countries lacking sufficient access to surgical care. The present report focuses on GTEMM efforts in Belize. Prior to the present study period, follow-up after surgical trips was conducted exclusively by local physicians in Belize City. However, given the common presentation of patients from distant locations throughout the country, post-operative follow-up was often poor. In an attempt to better understand methods of communication and optimize post-operative follow-up, we conducted a 2-part prospective investigation as part of visiting surgical trips to Belize. First, we aim to identify communication modalities available to patients in Belize to optimize post-operative follow-up. The second portion of our investigation used this data to test a pilot program for post-operative surgical follow-up using telephone interviews.

## Main text

### Methods

Three 1-week surgical trips providing urologic and urogynecologic surgical care to Belize were conducted by the GTEMM from 2014 to 2016. Mission work was conducted at the primary public hospital in Belize City (Karl Heusner Memorial Hospital). The majority of patients presented in a walk-in fashion resulting from radio and internet advertisements. Local providers also referred patients with complex urologic and urogynecologic pathology to the mission practitioners. Patients were examined and triaged by mission practitioners to operative or non-operative management. Appropriate patients underwent surgery and peri-operative recovery as in-patients. As part of this surgical experience, communication methods were assessed and a post-operative telephone surveillance initiative performed as described in the following sections.

#### Communication methods

All patients (n = 54) receiving evaluation by the GTEMM during a surgical trip in May 2014 completed a 6-item questionnaire. Both surgical and non-surgical patients completed questionnaire evaluation and no study exclusion criteria were used. The questionnaire focused on access to types of communication and preferred method of communication (Additional file 1: Appendix S1). In addition, patient demographics were collected which included age and residency/locality distance from Belize City. Residency population density was determined from published municipality data. Statistical analysis was performed via two-tailed Mann–Whitney U test to assess for a relationship between demographic variables and communication.

#### Post-surgical telephone surveillance

Based on our initial study results suggesting that mobile telephone was the most commonly utilized communication technology in this population, we instituted a pilot program to perform additional post-operative follow-up through telephone interview. This portion of the study was conducted during two subsequent mission trips in 2015–2016 and included only patients undergoing surgery (n = 35). Accordingly, all patients undergoing surgery were provided a discharge packet that included a specific date and time for 6-week post-operative telephone interview with a visiting physician located in the United States. Patients were instructed to call the visiting physician and were given a telephone card with 20 free minutes to minimize patient cost and facilitate compliance. Data regarding telephone surveillance were collected, including the compliance rate, length of telephone call, and whether any active surgical issues were identified that required further treatment.

Importantly, telephone surveillance was conducted in addition to directing patients to follow-up with local physicians at 2-weeks following surgery. This was done to improve compliance through multiple methods of follow-up and also to assess differences between these follow-up methods. Statistical analysis of data was performed using Student’s t test and Pearson’s analysis. Quantitative data are expressed as mean value (± SD). Each analysis was structured as a two-tailed test at the α = 0.05 level.

### Results

#### Communication methods

Fifty-four patients (n = 54) completed questionnaire evaluation. The mean patient age was 56 years (range 19–90) and 65% were female (35/54). Geographic data demonstrated that patients presented from a wide range of locations within Belize, coming from 5 of 6 districts throughout the country. Online populations statistics demonstrated a population density of these districts ranging from 17.1 to 58.7 (persons per square mile). Questionnaire outcomes are detailed in Additional file 1: Table S1. Patients reported the most access to mobile telephone, with 89% (48/54) responding that they had a mobile phone. There was a statistically significant relationship between patient age and access to home internet (p < 0.01) and email (p < 0.01), with younger patients having greater access to these communication methods. Residence population density did not demonstrate a statistical relationship with any form of communication access (p > 0.05).

#### Post-surgical telephone surveillance

A total of 35 patients underwent surgery during the trial period. Surgeries included a variety of major urologic and urogynecologic procedures, including nephrectomy, pelvic organ prolapse repair, pubovaginal sling placement, urethroplasty, transurethral resection of prostate and bladder tumor, and urethral diverticulectomy.

Eighteen (51%) patients were compliant with telephone interview at 6-week follow-up. Of these 18 patients, only 6 (33%) had been evaluated by a local physician following surgery. Three (17%) of 18 patients interviewed reported issues that required physician assistance. Two patients reported urinary straining after anti-incontinence surgery required evaluation with post-void residual. One patient required assistance obtaining pathology report and surveillance planning in the treatment of renal cancer.

A list of patients not compliant with phone interview was then provided to an in-country charity employee. Within the next 3 months, contact was achieved in 14/17 (82%) patients who were then seen in follow-up by a local physician. Three (21%) of these patients were contacted through secondary numbers (relative or friend) that had been obtained during initial evaluation.

The mean length of telephone interview was 8 min. The total cost of telephone minutes provided to patients was $175 USD, approximating $10 USD per compliant patient interview.

### Discussion

Achieving compliance with post-operative follow-up represents a significant challenge in LMIC. Previous literature has demonstrated poor rates of post-surgical follow-up in developing countries across a range of surgical specialties [7, 9, 10] For example, evaluation of a multi-center surgical experience in Asia and Africa details long-term (> 26 weeks) follow-up rates of 0% in half of the centers involved [10]. Barriers to post-operative follow-up in LMIC are well-described and include lack of transportation, cost associated with travel to medical facilities, and conflict with work or family responsibilities [9, 12, 13]. The importance of follow-up after surgery in the developing world is underscored by increased risk of surgical complication in this population as well as the improved outcomes and patient satisfaction that are associated with follow-up [14]. This challenge is even more significant in the context of visiting surgical trips given the often short-term nature of visiting services and lack of coordination with local practitioners.

Accordingly, research has focused on initiatives to improve follow-up using incentives as well as communication techniques. Travel subsidies, block appointments, and telephone reminders have all been shown to improve rates of follow-up after surgery [9–11]. More recently, programs using mobile phones have been used to improve follow-up after surgery in developing countries. Foong et al. reported improved compliance with speech therapy after cleft palate surgery in randomized study using a mobile phone program to facilitate follow-up logistics [4]. Patients randomized to receive a mobile phone after surgery demonstrated follow-up rates of 73% in comparison to 21% seen in patients not receiving a mobile phone. Limited reports are available to understand the efficacy of mobile phone technology in achieving follow-up specifically after international surgical trips. Using cellular phone interviews to improve compliance during cleft mission to rural Thailand, Wes and colleagues achieved follow-up in 54% of patients at 1.5 years post-operatively [6].

Our study reveals several notable findings. First, mobile phones are widely used in Belize. This finding supports numerous other reports demonstrating the significant adoption of mobile telephones throughout LMIC. Indeed, the majority of growth seen in mobile phone subscriber distribution over the last decade has been seen in the developing world [15]. Interestingly, age and resident population density did not demonstrate a statistical relationship with access to mobile telephones. This finding suggests a more widespread penetration of mobile technology and that mobile phones may be a valuable resource to improve surgical follow-up.

Second, our study demonstrated that phone interview may be a helpful method to increase compliance with follow-up. We were able to achieve initial telephone follow-up in over 50% of patients and the majority of these patients had not been compliant with their in-person follow-up appointment. Further, we found that the interviews conducted not only provided for subjective assessment of outcomes, but also identified issues that required evaluation and allowed for visiting physician assistance. Finally, given record of non-compliant patients at 6-week interview, our team was able to focus on these remaining patients and achieve an overall follow-up rate of 91%. We noted that several patients were ultimately reached through phone numbers of patient family or friends, highlighting the importance of recording secondary phone numbers during the initial triage visit.

In addition to the use of mobile phone technology, we believe comprehensive patient education during the mission trip was important to the success of our initiative. Indeed, prior research has demonstrated that an appropriate patient understanding of their disease and the importance of follow-up, as well as a detailed understanding of the follow-up plan, is fundamental to achieving compliance [4, 16]. Accordingly, our team focused on providing this information to patients throughout the surgical trip and supplied a detailed discharge packet with this information.

Our experience also identifies numerous areas for future investigation. First, it is important to understand the efficacy of telephone interview in assessing outcomes and complications after surgery. For example, validated questionnaires in assessment of prolapse and incontinence surgery are shown to correlate well with anatomic outcomes [17]. It is possible that the additional use of questionnaires during phone interview could provide improved assessment of patients undergoing these surgeries. Second, initiatives focused on patients at greatest risk for non-compliance may be helpful. Prior investigation demonstrates older age is associated with follow-up non-compliance, whereas patient education level and income are associated with compliance [9, 14]. Therefore, it may be possible to use these patient characteristics to identify groups at greater risk for non-compliance and introduce separate and more extensive compliance initiatives in this population.

### Conclusions

Phone interview may be a helpful method to increase compliance with follow-up in Belize and should be considered as an adjunct to local follow-up. Further, phone interview may serve as a valuable tool to assess outcomes during visiting surgical trips. Research is ongoing to further optimize post-operative follow-up during surgical trips.

### Limitations

Our experience is limited by patient number, which may influence results. In addition, these results are not generalizable to other international surgical trips given the individualized barriers and complexity that exist across different countries and surgical sites. In addition, it is possible that communication technologies and preferences differ across different countries.

## Additional file

**Additional file 1: Appendix S1.** Communication Methods, Questionnaire. **Table S1.** Demographics and Questionnaire Outcomes. English language version of questionnaire used for this study. Not previously published.

## Abbreviations

LMIC: low- and middle-income countries
GTEMM: Giving to Extremes Medical Missions
