# Supplementary material for: Assessment of communication technology and post-operative telephone surveillance during global urology mission
Source: BMC Res Notes. 2018 Feb 21;11:149. doi: 10.1186/s13104-018-3256-2 (PMC5822548; doi:10.1186/s13104-018-3256-2)
Supplement: Supplementary file 1 — Additional file 1: Appendix S1. Communication Methods, Questionnaire. Table S1. Demographics and Questionnaire Outcomes. English language version of questionnaire used for this study. Not previously published. [file 13104_2018_3256_MOESM1_ESM.docx]

**Appendix S1. Communication Methods, Questionnaire**

*1. Do you have a cell phone?*

*2. Do you have a home computer?*

*3. Do you have home internet?*

*4. Do you have an email account?*

*5. Do you have access to the internet outside of home (work or internet café)?*

*6. What is the best way to contact you regarding your health issues?*

**Table S1. Patient Demographics and Questionnaire Outcomes (n=54)**

*Age (y)(range)* 56 (19-90)

*Distance travelled to Clinic (miles)(range)* 35(5-176)

***Technology Access (%)***

*Mobile telephone* 48 (89)

*Home internet*  32 (59)

*Local internet*  28 (52)

*Email*  26 (48)

***Preferred Contact (%)***

*Mobile telephone* 40 (74)

*Email*  14 (26)
